# Myoinositol Supplementation of Freezing Medium Improves the Quality-Related Parameters of Dog Sperm

**DOI:** 10.3390/ani9121038

**Published:** 2019-11-27

**Authors:** Ahmad Yar Qamar, Xung Fang, Min Jung Kim, Jongki Cho

**Affiliations:** 1 College of Veterinary Medicine, Chungnam National University, Daejeon 34134, Korea; drahmadqamar@gmail.com (A.Y.Q.); fx2442@gmail.com (X.F.); 2 Department of Clinical Sciences, College of Veterinary and Animal Sciences, Jhang 35200, Sub-Campus University of Veterinary and Animal Sciences, Lahore 54000, Pakistan; 3 Department of Theriogenology and Biotechnology, College of Veterinary Medicine, Seoul National University, Seoul 08826, Korea

**Keywords:** myoinositol, dog sperm, cryo-survival, semen cryopreservation

## Abstract

**Simple Summary:**

The generation of free radical reactive oxygen species during freeze–thaw procedures is one of the major factors affecting the function and survival of sperm. Myoinositol is the most important natural form of inositol produced in the human body. Researchers have attempted to exploit the antioxidant nature of myoinositol to treat human infertility issues via the improvement of sperm quality traits and fertilization rates. We investigated the potential role of myoinositol neutralizing free radicals produced during the cryopreservation of dog semen. Myoinositol supplementation in the freezing medium resulted in improved quality-related parameters of dog semen including percentage motility, viability, plasma membrane integrity, and chromatin integrity. Improvement in post-thaw semen quality was confirmed by the expression of genes related to apoptosis, nuclear integrity, and reactive oxygen species generation.

**Abstract:**

Oxidative stress during freeze–thaw procedures results in reduced semen fertility. A decrease in free radical levels can improve the post-thaw sperm quality. We examined the effects of myoinositol supplementation in freezing medium on the structure and function of cryopreserved dog sperm. Pooled ejaculates were diluted with buffer without or with myoinositol (1 or 2 mg/mL). Analysis of fresh semen revealed that the optimal concentration of myoinositol was 1 mg/mL, and this concentration was used in further experiments. Post-thaw semen quality in the myoinositol-supplemented group was superior (*p* < 0.05) compared with that in the control group in terms of motility (57.9 ± 0.4% vs. 47.8 ± 0.2%), sperm viability (57.5 ± 0.5% vs. 44.6 ± 0.6%), intact plasma membrane (56.6 ± 0.4% vs. 46.2 ± 0.6%), and acrosome membrane (59.3 ± 0.5% vs. 51.8 ± 0.5%). In addition, sperm in the myoinositol-supplemented group showed a significantly lower expression of pro-apoptotic (*BAX*) and mitochondrial reactive oxygen species (ROS) modulator (*ROMO1*) genes but higher expression of anti-apoptotic (*BCL2*), and protamine-related (*PRM2* and *PRM3*) genes compared with that in the control group. Therefore, myoinositol supplementation before freezing can protect against oxidative stress and improve post-thaw dog sperm quality.

## 1. Introduction

Since its domestication, the dog (*Canis lupus familiaris*) has emerged as one of the major companion animals, playing an imperative role in human society [1]. The increasing socio-economic value of dogs has resulted in an increasing demand for advances in assisted reproductive technologies (ART) [2], particularly artificial insemination using cryopreserved semen [3]. Semen cryopreservation permits the exchange of genetic material among populations and preserves the genes of valuable individuals over extended periods [4,5]. In dogs, semen cryopreservation can help to conserve threatened breeds, enhance the use of certain genetic traits [6], and facilitate the breeding of individuals that are unable to breed naturally [7].

Despite the extensive use of cryopreserved semen in ART, the process of cryopreservation causes severe damage to the sperm membranes, mitochondria, DNA, as well as alters the functioning and metabolism [8]. These adverse effects are manifested in the form of impaired motility [9], decreased adenosine triphosphate (ATP) production [10], and reduced fertility [11]. The factors responsible for these deleterious changes include ice crystals formation, osmotic pressure disturbances, oxidative stress, and cholesterol efflux [12], as well as the loss of structural organization of the plasma membrane [13]. Oxidative stress is the result of uncontrolled systemic generation of reactive oxygen species (ROS), accompanied by the failure of the enzymatic system to detoxify them [14]. The absence of cytoplasmic defense systems and the presence of polyunsaturated fatty acids-rich membrane renders sperm more prone to oxidative damage [15]. However, ROS should not be completely eliminated because a certain level of ROS is required for normal reproductive functions including sperm capacitation, acrosome reaction, and fertilization [16]. Therefore, to achieve the optimal outcome following semen cryopreservation, efforts should focus on creating a balance between ROS production and elimination.

Myo-inositol (Myo-Ins), the naturally existing most active form of inositol, belongs to group 1 of the vitamin B complex. In the human body, Myo-Ins is mainly synthesized from glucose-6-phosphate [15]. Myo-Ins regulates the intracellular calcium (Ca^2+^) level by acting as a precursor for secondary messengers in the cellular signal transduction system [17]. In addition, Myo-Ins plays a vital role in different cellular functions including morphogenesis, cytogenesis, membrane formation, growth, and lipid production [18]. Human testicles contain high concentrations of enzymes, including Myo-Ins monophosphatase-1 and myoinositol-1-phosphate synthase, which are involved in the synthesis of Myo-Ins [19]. Bull testicular sperm can synthesize lipids from inositol that have an important role in providing essential compounds necessary for sperm survival in epididymis [20]. Myo-Ins regulates sperm maturation, motility, capacitation, and acrosomal reaction [21], as well as osmo-regulates the seminal plasma [22]. Furthermore, it improves sperm mitochondrial functions via Ca^2+^ ion influx that stimulates oxidation and ATP generation, thereby condensing chromatin and preventing apoptosis [19].

Researchers have attempted to exploit the antioxidant nature of Myo-Ins for treating human infertility issues, via the improvement of sperm quality-related parameters and fertility [23,24]. In-vitro Myo-Ins supplementation has been reported to effectively improve sperm quality in patients with oligoteratoasthenozoospermic (OAT) [19,25] as well as fertilization rate and embryo quality [24]. Inositol protects the enzyme systems from cryo-damage, lipid peroxidation [26], and preserves the acrosomal integrity of post-thaw ram sperm [27]. The aim of the present study was to investigate the effects of Myo-Ins supplementation on the different quality-related parameters of post-thaw dog semen by neutralization of ROS.

## 2. Materials and Methods

All chemicals used were purchased from Sigma-Aldrich (St. Louis, MO, USA) unless otherwise stated. Semen processing and evaluation was performed following the same protocol as previously explained [28].

### 2.1. Animals Used and Semen Collection

Four sexually mature and healthy male beagles, aged 2–4 years and weighing 8–11 kg, were used for semen collection. Separate indoor cages were used for each dog, furnished with all the necessary animal care facilities and procedures in accordance with the standards set up by the Committee for Accreditation of Laboratory Animals at Seoul National University. All experimental procedures were performed in compliance with the Guide for the Care and Use of Laboratory Animals at Seoul National University (approval no. SNU-180731-2). Semen was collected twice a week by digital manipulation. Ejaculates with a count of ≥ 100 × 10^6^ sperm/mL, ≥ 70% motile sperm, and ≥ 80% of viable sperm with normal morphology were pooled before use.

### 2.2. Determination of Optimal Myo-Ins Concentration

In Experiment 1, Myo-Ins was dissolved in buffer 1 [Tris (hydroxymethyl) aminomethane 24 g/L, citric acid 14 g/L, fructose 8 g/L, and kanamycin sulfate 0.15 g/L in distilled water (pH 6.6, 290 mOsm)] to achieve the Myo-Ins concentrations of 0, 1, or 2 mg/mL. The selection of Myo-Ins concentrations was decided on the basis of previously published work (1 mg/mL Myo-Ins, according to Ramadan Saleh 2018 [29]; 2 mg/mL Myo-Ins, according to Condorelli, 2011 [19]). The pooled ejaculates were washed via centrifugation at 100× *g* for 1 min at room temperature (25–28 °C), ensuring that debris descended to the bottom of the tube, and sperm remained in the supernatant [30]. The supernatant was divided into three equal aliquots and centrifuged at 700× *g* for 5 min at room temperature (25–28 °C). The final concentration of 200 × 10^6^ sperm/mL was achieved by separately resuspending each sperm pellet using buffer 1 with different Myo-Ins concentrations. The optimal Myo-Ins concentration was determined following the same procedure as described previously [31].

Sperm viability, motility, and kinematic parameters were assessed for each Myo-Ins concentration [30]. A drop of semen (10 µL) was placed onto a clean pre-warmed glass slide and mounted with a coverslip. Kinematic parameters were assessed by screening five different fields and tracking at least 200 motile sperm in each experiment using a sperm analysis imaging system (FSA2011 premium edition version 2011; Medical Supply, Gwangwon, Korea) [31,32].

Sperm viability was assessed using eosin-nigrosin staining. Briefly, sperm stain suspension was prepared by mixing a drop of semen (10 µL) in an equal volume of stain. Smears were made by spreading a drop of suspension on a clean glass slide followed by air drying. At least 200 sperm per slide were examined to assess membrane integrity; the membranes were classified as intact (white color) or damaged (pink color). The optimal Myo-Ins concentration was selected on the basis of outcomes of the analysis of fresh semen and was used for the remaining experiments.

### 2.3. Semen Cryopreservation and Thawing

Experiment 2 was performed to investigate the effects of Myo-Ins on sperm function and survival during cryopreservation. Pooled ejaculates were washed and diluted following the protocol described above. Sperm suspension was divided into two aliquots and diluted with buffer 2 [54% (*v/v*) buffer 1, 40% (*v/v*) egg yolk, and 6% (*v/v*) glycerol] supplemented with Myo-Ins; 0, or 1 mg/mL. Buffer 2 was added to the sperm suspension following a multistep dilution protocol (14%, 19%, 27% and 40% of total calculated volume, sequentially loaded at an interval 30 s) [28] until a final concentration of 100 × 10^6^ sperm/mL was achieved. The diluted semen was filled into 0.5-mL semen straws (Minitube, Tiefenbach, Germany) and equilibrated at 4 °C for 45–60 min. Semen straws were placed horizontally 2 cm above the surface of liquid nitrogen (LN_2_) for 15 min and finally plunged into the LN_2_ for storage.

After 1 week of storage, frozen semen was thawed in a water bath at 37 °C for 30 s and diluted (1:5) with buffer 1 to 14%, 19%, 27%, and 40% of the total volume [32]. Progressive sperm motility, kinematic parameters, and viability were assessed after 5 min of thawing following the protocol described in ‘Section 2.2’.

### 2.4. Assessment of Sperm Plasma Membrane Integrity

Hypo-osmotic swelling (HOS) assay was performed to analyze sperm plasma membrane integrity [33]. A drop of semen (50 µL) from each group was mixed in 0.5 mL of HOS solution and incubated at 37 °C for 30 min. A 5-µL drop of the mixture was placed on a pre-warmed clean glass slide, and 200 sperm were examined within 5–10 min for their ability to swell using phase-contrast microscopy (Eclipse Ts2, Nikon, Minato-Ku, Tokyo, Japan). The swelling was indicated by the coiling of the sperm tail and such sperm were considered to possess an intact plasma membrane.

### 2.5. Assessment of Sperm Acrosome Membrane Integrity

Sperm acrosome membrane was analyzed using fluorescein isothiocyanate-conjugated peanut agglutinin (FITC-PNA) [34]. Post-thaw semen was smeared on glass slides and air-dried. Smears were fixed for 10 min at 20 °C–22 °C using absolute methanol, followed by air drying. Staining was performed by spreading 30-µL of FITC-PNA solution [100 µg/mL of phosphate buffer saline (PBS)] on the slides, followed by incubation at 37 °C for 30 min in the dark under humid conditions. Finally, the slides were rinsed with PBS, dried, and mounted with glycerol. The status of sperm acrosomes was analyzed using epifluorescence microscopy (Eclipse Ts2, Nikon) and classified as intact acrosome (strong green fluorescence), partially intact acrosome (dull green fluorescence), or non-intact acrosome (no fluorescence).

### 2.6. Mucus Penetration Test

Surrogate mucus (modified synthetic oviductal fluid) [28,30] was loaded into flat capillary tubes (80 ± 0.5 mm long, 1.25 ± 0.05 mm wide, Hilgenberg GMBH, Stutzerbach, Germany) sealed at one end. The capillary tubes were placed in the vertical position for 15 min to check the tightness of the seal and to remove bubbles following which the open end of the capillary tube was inserted into an Eppendorf tube containing 100 µL of semen suspension and placed horizontally for 2 h at room temperature (25–28 °C). Thereafter, the number of sperm that reached the 1 and 3 cm marks in the capillary tube was counted.

### 2.7. Protamine Deficiency Test

**Figure 1:**
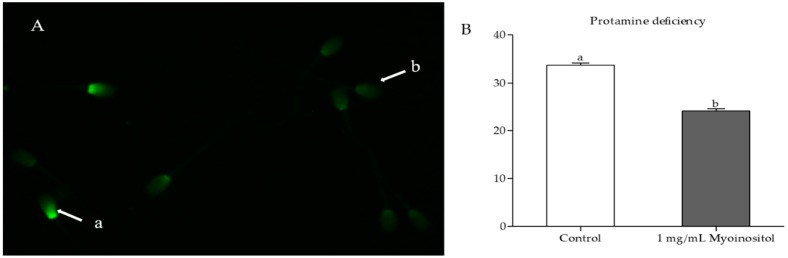
(**A**) Chromomycin A_3_ (CMA_3_) staining of sperm chromatin to demonstrate protamine-deficient (**a**) and normal sperm (**b**). (**B**) Quantification of CMA_3_ in myoinositol-treated and untreated sperm (*p* < 0.05).

Chromomycin A_3_ (CMA_3_) staining was performed to examine the sperm chromatin packing [35]. Post-thaw sperm were washed using buffer 1 and smeared onto clean glass slides. Smears were fixed in methanol: Glacial acetic acid (3:1) for 5 min at 4 °C and treated with 100 µL of CMA_3_ solution (0.25 mg/mL of McI1vaine buffer supplemented with 10 mM MgCl_2_, pH 7.0) for 20 min. Smears were rinsed in McI1vaine buffer [30], mounted with buffered glycerol, and examined using epifluorescence microscopy (Eclipse Ts2, Nikon). CMA_3_-positive sperm with abnormal chromatin packing were indicated by their bright green fluorescent heads, whereas CMA_3_-negative sperm with normal chromatin packing were indicated by their dull green fluorescent heads (Figure 1A).

### 2.8. Assessment of Gene Expression

Quantitative polymerase chain reaction (qPCR) was used to analyze the messenger ribonucleic acid (mRNA) expression of genes related to apoptosis (B-cell lymphoma (*BCL2*), BCL2-associated X (*BAX*)), and protamine level measurement [protamine 2 (*PRM2*) and protamine 3 (*PRM3*)], as well as mitochondrial ROS modulator (ROS modulator 1 (*ROMO1*)). Briefly, RNA was extracted from post-thaw sperm both from the Myo-Ins-supplemented and control groups. Real-time qPCR (RT-qPCR) was used for assessing transcript abundance (primers listed in Table 1). Trizol reagent (Invitrogen, Carlsbad, CA, USA) was used for RNA extraction, following which complementary DNA synthesis was performed using the Compact cDNA synthesis kit (SJ BIOSCIENCE, Daejeon, Korea), according to the manufacturer’s instructions. Expression levels of RT-qPCR transcripts were analyzed using the Sybr Green Q-PCR Master Mix (SJ BIOSCIENCE, Daejeon, Korea) and the expression of each target gene was quantified relative to that of the internal gene β-actin (*ACTB*) using the equation, R = 2^–[ΔCt sample − ΔCt control]^.

### 2.9. Statistical Analysis

Data analysis was performed using SPSS 21.0 software (SPSS Inc., Chicago, IL, USA). All values are expressed as mean ± SEM, and *p* < 0.05 indicated statistical significance. Data related to the determination of optimal Myo-Ins concentration were analyzed using a one-way analysis of variance and Tukey’s multiple comparison test. The independent sample *t*-test was used to compare different quality-related parameters and gene expression between the post-thaw semen samples of the Myo-Ins-supplemented and control groups.

## 3. Results

### 3.1. Determination of Optimal Myo-Ins Concentration

The percentage motility of fresh sperm supplemented with 2 mg/mL Myo-Ins was significantly reduced (64.7% ± 0.7%) compared with that of the control and sperm supplemented with 1 mg/mL (73.8% ± 0.3% and 71.5% ± 0.4%, respectively; Table 2). The percentage linearity observed in both 1 and 2 mg/mL Myo-Ins-supplemented semen samples was similar (27.0% ± 0.5%, and 25.7% ± 0.4%) but higher than that in the control (24.1% ± 0.3%). The amplitude of lateral head displacement (ALH) was significantly lower in the 2 mg/mL Myo-Ins-supplemented semen samples (4.1% ± 0.1%) compared with that in the others (5.0% ± 0.1% for the control and 4.6% ± 0.1% for 1 mg/mL). Straightness (%) did not significantly differ among the groups.

The effects of Myo-Ins supplementation on fresh sperm viability were evaluated following the examination of four independent replicates. The difference in live sperm percentages was not significant between the control and 1 mg/mL Myo-Ins-supplemented semen samples (69.2% ± 0.2% and 68.7% ± 0.2%, respectively), but both of these differences were significantly higher than 2 mg/mL Myo-Ins-supplemented semen samples (61.2% ± 0.3%).

### 3.2. Effect of Myo-Ins on Post-Thaw Sperm Motility and Kinematic Parameters

The post-thaw analysis performed after 5 min of thawing revealed that the motile sperm percentage was significantly greater in Myo-Ins-supplemented sperm samples (57.9% ± 0.4%) compared with that in the control (47.8% ± 0.2%; Table 3). The kinematic parameters exhibited similar trends as observed for percentage motility among the samples of Myo-Ins-supplemented and control groups. The percentages of linearity, straightness, and ALH were significantly higher in Myo-Ins-supplemented sperm samples than those in the control (29.6% ± 1.1% vs. 23.9% ± 0.3%, 54.5% ± 0.6% vs. 50.8% ± 0.9%, and 3.3% ± 0.0% vs. 2.5% ± 0.0%; respectively), (Table 3).

### 3.3. Effect of Myo-Ins on Sperm Viability and Plasma Membrane Integrity

Eosin-nigrosin staining of post-thaw sperm showed that the percentage of live sperm was significantly higher in Myo-Ins-supplemented samples than that in the control (57.5% ± 0.5% vs. 44.6% ± 0.6%, respectively; Table 3). Further investigations revealed that improvement in sperm plasma membrane integrity contributed to higher live sperm percentages. HOS assay showed Myo-Ins-supplemented sperm samples had a significantly higher percentage of intact plasma membrane (56.6% ± 0.4%) compared with the control (46.2% ± 0.6%).

### 3.4. Effect of Myo-Ins on Sperm Acrosome Integrity

FITC-PNA staining revealed that Myo-Ins supplementation resulted in a significantly higher percentage of sperm with intact acrosome compared with the control (59.3% ± 0.5% vs. 51.8% ± 0.5%; respectively; Table 4).

### 3.5. Effect of Myo-Ins on Mucus Penetration

Mucus penetration test was performed to examine the effect of Myo-Ins supplementation on the ability of sperm to penetrate mucus. Post-thaw analysis showed that Myo-Ins-supplemented sperm penetrated the synthetic oviductal fluid more effectively. The sperm counts at both the 1 and 3 cm marks were significantly higher for Myo-Ins-supplemented sperm samples (150.4 ± 0.6 sperm and 57.0 ± 0.4 sperm, respectively) than those for the control group (139.6 ± 0.5 sperm and 48.1 ± 0.7 sperm, respectively; Table 4).

### 3.6. Effect of Myo-Ins on Chromatin Integrity

CMA_3_ staining was used to assess the quality of chromatin packing in post-thaw sperm. Samples that received 1 mg/mL Myo-Ins supplementation (group 1) during freezing showed a significantly lower proportion of sperm with protamine deficiency after thawing compared with the samples in the control group (24.1% ± 0.5% vs. 33.6% ± 0.5%; Figure 1B).

### 3.7. Effect of Myo-Ins on Gene Expression

**Figure 2:**
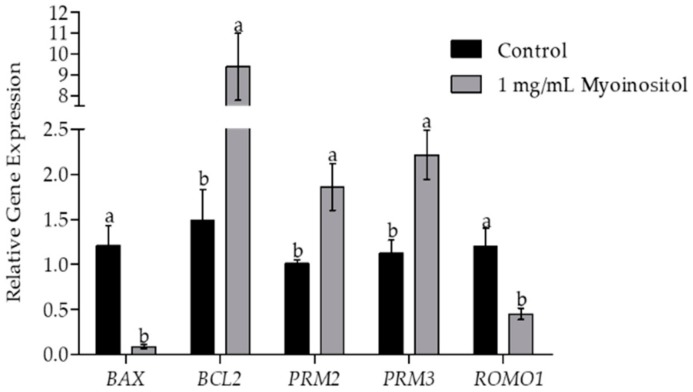
Expression of the pro-apoptotic gene *BCL2*-associated X, (*BAX*); the anti-apoptotic gene B-cell lymphoma (*BCL2)*; the chromatin repair-related genes protamine-2 (*PRM2*) and protamine-3 (*PRM3*); and the mitochondrial ROS modulator gene (*ROMO1*) using RT-qPCR in myoinositol-supplemented and non-supplemented sperm samples. Values are presented as mean ± SEM. Different lowercase letters (a or b) represent a significant difference (*p* < 0.05).

Analysis of expression levels of different genes in post-thaw semen samples was performed by RT-qPCR. The effects of the supplementation of buffer 2 with 1 mg/mL Myo-Ins on apoptosis-related genes were exhibited by the significantly enhanced expression levels of *BCL2* and reduced expression of *BAX* compared with the control group (Figure 2). The expression levels of genes associated with protamine levels (*PRM2* and *PRM3*) in post-thaw semen samples were significantly higher in Myo-Ins-supplemented semen samples than in the control group. Furthermore, the expression levels of the mitochondrial ROS modulator (*ROMO1*) were significantly reduced in Myo-Ins-supplemented sperm samples compared with the control.

## 4. Discussion

Semen cryopreservation is an essential part of ART. However, freeze–thaw procedures are associated with oxidative stress that causes reductions in sperm function [36], cryo-survival [37], and fertility [38,39,40]. Oxidative stress is a result of increased ROS generation and decreased antioxidant levels that render sperm more susceptible to lipid peroxidation [41]. To reduce the magnitude of oxidative damage during dog semen cryopreservation, we supplemented buffer 2 with Myo-Ins, a natural sugar produced in the human testicles that protects against ROS by improving membranal integrity, mitochondrial function, and chromatin compactness as well as preventing apoptosis [19]. To reduce the direct effects of Myo-Ins on metabolism in sperm, we determined the optimal Myo-Ins concentration using fresh dog semen. In the group supplemented with 2 mg/mL Myo-Ins, sperm motility, ALH, and viability were significantly reduced (Table 2), possibly due to a change in osmotic pressure or toxicity caused by the Myo-Ins. The optimal Myo-Ins concentration observed in this study was 1 mg/mL similar to that reported in for humans [29].

Results showed that supplementation of semen extender (buffer 2) with 1 mg/mL of Myo-Ins had beneficial effects on the post-thaw dog sperm motility (Table 3). In the middle piece of the sperm tail, the axosome and associated dense fibers are surrounded by mitochondria serving as a powerhouse for sperm by generating ATP [42]. It is well-known that ROS damage the axosome and mitochondria, resulting in sperm immobilization [43]. Sperm with disrupted membrane integrity exhibit high membrane permeability and are unable to control intracellular ion concentrations responsible for its motion [44]. The post-thaw results suggest that Myo-Ins supplementation significantly enhances dog sperm motility, membrane integrity (plasma and acrosome), and viability (Table 3 and Table 4) indicating the protective nature of Myo-Ins against cryo-damage due to oxidative stress. Myo-Ins improves mitochondrial function, affecting ATP generation in patients with altered semen quality [19]. Incubation of fresh human semen with Myo-Ins (2 mg/mL) resulted in a significant improvement of motility [25]. Similarly, the positive influence of Myo-Ins on motility of fresh and post-thaw human [15,45], and bovine [46,47].

Decreased levels of *ROMO1* (a mitochondrial ROS modulator) in Myo-Ins-supplemented sperm indicate improved mitochondrial function. *ROMO1* is the main gene involved in the generation of ROS from mitochondria [48], which is responsible for the programmed death of cells [49]. The decreased expression of the *BAX* (pro-apoptotic gene) along with increased expression of the *BCL2* (anti-apoptotic gene) observed in this study indicate reduced apoptosis during sperm cryopreservation (Figure 2). Post-thaw analysis of Myo-Ins-supplemented semen samples confirmed significantly higher cryo-survival compared with the control (Table 3). Similarly, Myo-Ins supplementation (1 mg/mL) of the freezing medium reportedly results in a significant improvement in the survival rate of human sperm [29].

Sperm chromatin packing involves the replacement of the original histone protein with protamine [50,51]. The large segments of DNA have a stronger bond with protamine than with histone [52] and the resultant condensed strands of DNA (toroids) have better protection against the oxidative damage of chromatin [53]. The conservation of sperm protamine content during freeze–thaw indicates the integrity of sperm chromatin material. Post-thaw sperm analysis showed significantly lower percentages of protamine-deficient sperm in the Myo-Ins-supplemented group compared with those in the control (Figure 1). Transcript analysis of Myo-Ins supplemented semen samples showed a significantly higher expression of protamine genes (*PRM2* and *PRM3*) compared with that in the control (Figure 2). Myo-Ins treatment has been shown to significantly enhance the integrity of human sperm chromatin by reducing DNA fragmentation [15]. Similarly, the supplementation of Myo-Ins prior to semen processing protects the integrity of bovine sperm DNA [47].

The mucus penetration test reflects the ability of sperm to pass through the cervical mucus barrier. It serves as a useful tool for measuring the migratory capability of sperm through the genital tract [54]. Additionally, the sperm’s ability to penetrate the cervical mucus surrogates is closely correlated with its ability to fuse with oocytes [55]. In the present study, a significantly higher number of sperm reached the 1 and 3 cm distance in the surrogate cervical mucus (synthetic oviductal fluids) in the Myo-Ins-supplemented sperm group. Improved mucus penetration ability and significant ALH increase indicate sperm hyperactivation, which can result in enhanced fertilization and pregnancy rates [56].

## 5. Conclusions

Myo-Ins (1 mg/mL) supplementation of buffer 2 (the extender) can provide protection to dog sperm during cryopreservation, improve the post-thaw sperm motility, kinematic parameters, membrane integrity, and reduce chromatin damage and apoptosis. However, further studies are required to determine the impact of Myo-Ins on in-vivo fertilization and pregnancy rates.

## Figures and Tables

**Table 1:** Primer sequences used for gene expression analysis.

Gene	Primer Sequence (5′–3′)	Product Size (bp)	NCBI Accession No.
*BACT*	F: GAGGCATCCTGACTCTGA	87	XM_544346.3
	R: TCTGGCACCACACTTTCT		
*BCL2*	F: GACAGAGAGGATCATGCTGT	141	NM_001002949.1
	R: TGGCATGAGATGCAGGAAAT		
*BAX*	F: CCAAGAAGCTGAGCGAATG	123	NM_001003011.1
	R: CTGCCACTCGGAAGAAGAC		
*PRM2*	F: CTCCAGAAGGGTCAGGAG	169	NM_001287148.1
	R: GGCTCCTTGCAAACTCAG		
*PRM3*	F: TCTGGAGAGGCAGCCAGA	101	XM_022420065.1
	R: AGGCCATGAGCTTCTTCA		
*ROMO1*	F: CTACGTGCTCCCGGAAGT	100	XM_534406.6
	R: TCGCTCAGTTCTACGTCTCAC		

F, forward; R, reverse; *BCL2*, B-cell lymphoma; *BAX*, *BCL2*-associated X; *PRM2*, protamine 2; *PRM3*, protamine 3; *ROMO1*, reactive oxygen species modulator 1.

**Table 2:** Determination of optimal Myo-Ins concentration for dog semen cryopreservation.

Group	Motility (%)	Linearity (%)	Straightness (%)	ALH (µm)	Live Sperm (%)
0 mg/mL (control)	73.8 ± 0.3 ^a^	24.1 ± 0.3 ^b^	48.4 ± 0.5	5.0 ± 0.1 ^a^	69.2 ± 0.2 ^a^
1 mg/mL	71.5 ± 0.4 ^b^	25.7 ± 0.4 ^a,b^	49.5 ± 0.5	4.6 ± 0.1 ^a^	68.7 ± 0.2 ^a^
2 mg/mL	64.7 ± 0.7 ^c^	27.0 ± 0.5 ^a^	49.5 ± 0.9	4.1 ± 0.1 ^b^	61.2 ± 0.3 ^b^

ALH, amplitude of lateral head displacement. ^a,b^ Values in columns with different superscript lowercase letters significantly differ (*p* < 0.05, *n* = 4).

**Table 3:** Effect of Myo-Ins supplementation on post-thaw dog semen quality after 5 min of thawing.

Group	Motility (%)	Linearity (%)	Straightness (%)	ALH (µm)	Live Sperm (%)	Membrane Integrity (%)
Control (0 mg/mL)	47.8 ± 0.2 ^b^	23.9 ± 0.3 ^b^	50.8 ± 0.9 ^b^	2.5 ± 0.0 ^b^	44.6 ± 0.6 ^b^	46.2 ± 0.6 ^b^
Treatment (1 mg/mL)	57.9 ± 0.4 ^a^	29.6 ± 1.1 ^a^	54.4 ± 0.6 ^a^	3.3 ± 0.0 ^a^	57.5 ± 0.5 ^a^	56.6 ± 0.4 ^a^

ALH, amplitude of lateral head displacement. ^a,b^ Values in columns with different superscript lowercase letters significantly differ (*p* < 0.05, *n* = 4).

**Table 4:** Effects of supplementing buffer 2 with Myo-Ins on post-thaw dog semen quality.

Group	Acrosome Integrity (%)	Sperm Count at Mucus Penetration Distance	
		1 cm	3 cm
Control	51.8 ± 0.5 ^b^	139.6 ± 0.5 ^b^	48.1 ± 0.7 ^b^
Treatment (1 mg/mL)	59.3 ± 0.5 ^a^	150.4 ± 0.6 ^a^	57.0 ± 0.4 ^a^

^a,b^ Values in columns with different superscript lowercase letters significantly differ (*p* < 0.05, *n* = 4).
